# Evolution and variability of *Solanum RanGAP2,* a cofactor in the incompatible interaction between the resistance protein GPA2 and the *Globodera pallida* effector Gp-RBP-1

**DOI:** 10.1186/1471-2148-13-87

**Published:** 2013-04-19

**Authors:** Jean Carpentier, Eric Grenier, Magalie Esquibet, Louis-Philippe Hamel, Peter Moffett, Maria J Manzanares-Dauleux, Marie-Claire Kerlan

**Affiliations:** 1 INRA, UMR 1349 IGEPP INRA, Agrocampus Ouest, Université Rennes1, Ploudaniel, Keraïber, F.29260, France; 2 Agrocampus Ouest, UMR 1349 IGEPP INRA, Agrocampus Ouest, Université Rennes1, Le Rheu, F-35653, France; 3 INRA, UMR 1349 IGEPP INRA/Agrocampus Ouest/Université Rennes1, Le Rheu, F-35653, France; 4 Université Sherbrooke, Dept Biol, Sherbrooke, PQ, J1K 2R1, Canada; 5 Université Européenne de Bretagne, Brittany, Rennes cedex, F-35042, France

## Abstract

**Background:**

The Ran GTPase Activating Protein 2 (RanGAP2) was first described as a regulator of mitosis and nucleocytoplasmic trafficking. It was then found to interact with the Coiled-Coil domain of the Rx and GPA2 resistance proteins, which confer resistance to Potato Virus X (PVX) and potato cyst nematode *Globodera pallida*, respectively. RanGAP2 is thought to mediate recognition of the avirulence protein GP-RBP-1 by GPA2*.* However, the *Gpa2-*induced hypersensitive response appears to be relatively weak and *Gpa2* is limited in terms of spectrum of efficiency as it is effective against only two nematode populations. While functional and evolutionary analyses of *Gp-Rbp-1* and *Gpa2* identified key residues in both the resistance and avirulence proteins that are involved in recognition determination, whether variation in *RanGAP2* also plays a role in pathogen recognition has not been investigated.

**Results:**

We amplified a total of 147 *RanGAP2* sequences from 55 accessions belonging to 18 different di-and tetraploid *Solanum* species from the section Petota. Among the newly identified sequences, 133 haplotypes were obtained and 19.1% of the nucleotide sites were found to be polymorphic. The observed intra-specific nucleotide diversity ranges from 0.1 to 1.3%. Analysis of the selection pressures acting on *RanGAP2* suggests that this gene evolved mainly under purifying selection. Nonetheless, we identified polymorphic positions in the protein sequence at the intra-specific level, which could modulate the activity of RanGAP2. Two polymorphic sites and a three amino-acid deletion in RanGAP2 were found to affect the timing and intensity of the *Gpa2*-induced hypersensitive response to avirulent GP-RBP-1 variants even though they did not confer any gain of recognition of virulent GP-RBP-1 variants.

**Conclusions:**

Our results highlight how a resistance gene co-factor can manage in terms of evolution both an established role as a cell housekeeping gene and an implication in plant parasite interactions. St*RanGAP2* gene appears to evolve under purifying selection. Its variability does not seem to influence the specificity of GPA2 recognition but is able to modulate this activity by enhancing the defence response. It seems therefore that the interaction with the plant resistance protein GPA2 (and/or Rx) rather than with the nematode effector was the major force in the evolution of the RanGAP2 locus in potato. From a mechanistic point of view these results are in accordance with a physical interaction of RanGAP2 with GPA2 and suggest that RBP-1 would rather bind the RanGAP2-GPA2 complex than the RanGAP2 protein alone.

## Background

Wild plant genetic resources are often used in plant breeding strategies to improve various agronomical traits of cultivated plants. Wild plants are continuously evolving to adapt to their changing environmental conditions such as climatic changes, soil quality or pathogens attacks. One consequence of the long evolution of wild species is the diversity observed for some phenotypic characteristics. This diversity is reflected in the genetic variability at the loci controlling the trait. Thus, wild species represent a large pool of valuable diversity that is useful for transferring new agriculturally-important traits, such as pathogen resistance, to crop plants.

Knowledge of the diversity and evolutionary dynamics of genes involved in host-pathogen interactions is needed to generate resistant varieties as well as to prevent pathogens from overcoming resistance. In plant-parasite interactions, recognition of pathogen effectors results in the activation of effector triggered immunity (ETI), in which case the effector is often referred to as an avirulence (Avr) protein [1]. Intracellular receptors involved in ETI are usually encoded by resistance (*R*) genes most of which belong to the nucleotide-binding leucine-rich repeat protein family (NB-LRR) [2]. Many plant-pathogen interactions can be explained by the “gene-for-gene” model in which an *R* gene provides resistance to the pathogen carrying the corresponding *Avr* gene [3,4]. Avr proteins can activate R proteins directly or via a cofactor that interacts with the Avr protein [5]. Indirect interactions are often described by the Guard [4,6] or Decoy [7] models. These models predict that R proteins act by monitoring the status of effector targets also known as guardees or decoys. In the absence of the *R* protein, the targeting of the guardee enhances pathogen virulence whereas the decoy has no impact on virulence or pathogen fitness, but simply resembles the actual virulence target of the effector. While such cofactors appear to play a role in the recognition of the *Avr* protein [1,6], they likely also function in the proper maturation of the R protein [5].

Among the identified NB-LRR cofactors, few have been investigated in terms of sequence variability and evolutionary forces shaping their diversity in plant species. The combined analysis of 27 *R* genes and 27 downstream genes in *Arabidopsis*[8,9] revealed that although R genes showed hallmarks of balancing selection or partial selective sweeps, most of defence response genes appear to be under purifying selection. The correlation between evolutionary rates and the position at which a gene operates in a pathway has been recently studied using the *Pto/Prf* signalling pathway in tomato [10]. This pathway involves one NB-LRR gene (*Prf*), two upstream co-factors (*Pto* and *Fen*) and a third co-factor (*Rin4*) that is predicted to activate downstream defences in tomato, but is also known for its co-factor function in other signalling pathways like the *Rpm1* pathway in *Arabidopsis*[11,12]. Although *Pto* showed a clear signature of balancing selection, *Rin4* showed predominantly purifying selection which tend to suppress the apparition of new variants and *Fen* lies between these two genes with signatures of both balancing and purifying selection. More recently, allelic diversity at the locus encoding the RCR3 protein, guarded by R protein Cf2 has been shown to be maintained by balancing selection in the wild tomato species *Solanum peruvianum*. Guardee evolution might be governed by a counterbalance between improved activation in the presence of the corresponding pathogen and prevention of auto-immune responses in its absence [13].

RanGAP proteins were first described as cytoplasmic proteins involved in mitosis and nucleocytoplasmic trafficking [13-15]. The potato RanGAP2 protein has also been described as a co-factor of R proteins, since it physically interacts, through its WPP domain, with two *Solanum tuberosum* resistance proteins called Rx and GPA2 [16]. Rx and GPA2 confer resistance to PVX and potato cyst nematode *Globodera pallida*, respectively [17] whereas *Gpa2* confers resistance to a limited number of *G. pallida* populations [18]. Compared to Rx, Gpa2 induces a much weaker hypersensitive response (HR) when transiently expressed with its effector counterpart (the avirulence protein Gp-RBP-1 of *G. pallida*) in tobacco [19]. Unexpectedly, numerous variants of the *G. pallida* avirulence protein GP-RBP-1 are recognized by GPA2, even those from nematode populations described as virulent against *S. tuberosum* cultivars expressing *Gpa2*[20]. Thus, other proteins involved in the recognition of GP-RBP-1 by GPA2 may explain these results and RanGAP2 has been thought to mediate recognition of the avirulence protein GP-RBP-1 by GPA2. Potential for variation in effector recognition due to variation in bait proteins [5] is suggested from studies of the tomato NB-LRR Prf, which recognizes Avr proteins from *Pseudomonas syringae* through its cofactor, the Pto kinase [21]. Moreover, artificial tethering experiments also suggested that RanGAP2 may facilitate the recognition of Avr proteins via the LRR domains of Rx and GPA2 [19].

In potato, tobacco and Arabidopsis, two RanGAP proteins (RanGAP1 and RanGAP2) have been described. RanGAP1 binds weakly or not at all to Rx *in planta* and is therefore probably not important for the function of Rx/GPA2 [22]. Although the exact mechanism of recognition of the Avr protein by Rx and GPA2 is still unclear, previous studies have shown that RanGAP2 is required for Rx and GPA2 function [16]. The genetic diversity of the genes encoding both the avirulence proteins (PVX CP and Gp-RBP-1) and the resistance proteins (Rx and GPA2) has been well characterized. A high frequency of polymorphic sites in the four genes has been identified, including key residues necessary for recognition of the CP and GP-RBP-1 by Rx and GPA2, respectively [19,20,23,24]. On the other hand, the genetic diversity or sequence polymorphism of the co-factor *RanGAP2* has not yet been reported. To understand the potential role of potato RanGAP2 in the recognition of Gp-RBP-1 by GPA2 and to eventually identify potato *RanGAP2* variants able to enhance or enlarge the spectrum of recognition of *Gpa2* towards *G. pallida* populations, it is necessary to determine the molecular variability and the evolutionary dynamics of this key co-factor.

In this context, the aims of this study were to: i) evaluate the genetic diversity of *RanGAP2* in different *Solanum* species and ii) understand how *RanGAP2* variability affects the GPA2-mediated immune response. To this end, we explored *RanGAP2* variability in 55 accessions belonging to 18 *Solanum* species, looking for selective pressure hallmarks and key polymorphic positions in *RanGAP2*, which could potentially affect either the intensity of the HR or recognition specificity of Gp-RBP-1 variants by GPA2.

## Results

### *RanGAP2* structure and variability

To amplify the *RanGAP2* gene from *Solanum* species we designed primers based on the *Solanum RanGAP2* sequence originating from *S. tuberosum* cv Desiree [http://www.ncbi.nlm.nih.gov/nuccore/AM411448]. Comparison of *RanGAP1* [http://www.ncbi.nlm.nih.gov/nuccore/AM411449.1] and *RanGAP2* gene sequences from the same cultivar (Desiree) showed that they are only 66% identical while the *RanGAP2* sequences from *S. tuberosum* and *Nicotiana benthamiana* [http://www.ncbi.nlm.nih.gov/nuccore/EF396237.1] are 91% identical, indicating strong conservation of *RanGAP2* sequences in the *Solanaceae* family. We amplified a total of 147 *RanGAP2* sequences from 55 accessions belonging to 18 different *Solanum* species from the section Petota. The maximum number of allelic variants obtained for each genotype corresponded to its ploidy level (two for the diploids and four for the tetraploids), indicating that *RanGAP2* is most likely a single-copy gene in the *Solanum* genome. BLAST searches against the GenBank/EMBL Databases gave best hits with *RanGAP2* (more than 97% identity with an e-value of 0) and *RanGAP1* [AM411449.1] (73% identity on average with an e-value lower than 10^-89^), suggesting that, apart from *RanGAP1*, *RanGAP2* does not have any close homologues in potato. All the *RanGAP2* sequences obtained lacked introns and had a CDS of 1662 bp (553 amino acids), with the exception of one sequence obtained from accession 78S.248.1 of *S. vernei* (sequence VRN2-A1). This particular sequence variant has a nine nucleotide in frame deletion (nucleotides 1546 to 1554 corresponding to amino acids 516 to 518). All sequences encoded for a WPP domain at the 5’end (corresponding to amino acids 14 to 109) and 10 LRRs (corresponding to amino acids 213 to 493), a structure identical to that of the *RanGAP2* orthologs sequenced in *N. tabacum* and *Arabidopsis thaliana*. Polymorphism analysis revealed 318 (19.1%) polymorphic sites spread over the entire *RanGAP2* sequence: 162 singletons and 156 parsimony informative sites. Among these 318 mutations, 47% are non-synonymous (mutations causing amino acid substitutions). Furthermore, 67% and 55% of the singletons and parsimony informative sites, respectively, are non-synonymous. One hundred and thirty three haplotypes were obtained from the 147 sequences. Haplotypes shared between species: *S. tuberosum* ssp *andigena*/*S. phureja*, *S. tuberosum* ssp *tuberosum*/*S. vernei* and *S. vernei*/*S. spegazzinii* (Table 1). The pi diversity index of most tetraploid species was higher than that of the diploid species. The exception was the cultivated diploid species *S. stenotomum,* which showed the highest sequence diversity.

**Table 1:** **Nucleotide and haplotype variability within the 18 *Solanum *species studied**

**Species**	**Wild / cultivated**	Genome formula*	**# of acc.**	**# of seq.**	**pi**	**# of haplotypes**	**# of segregating sites**	**D Tajima**	**% NS mutations**
*S. berthaultii*	*wild*	AA	2	4	0.0019	4	6	-0.314	33.30%
*S. trifidum*	*wild*	BB	2	3	0.0020	3	5	ND	40.00%
*S. chacoense*	*wild*	AA	2	2	0.0024	2	4	ND	25.00%
*S. kurtzianum*	*wild*	AA	1	2	0.0024	2	4	ND	25.00%
*S. tarijense*	*wild*	AA	2	3	0.0028	2	7	ND	71.40%
*S. phureja*	*cultivated*	AA	2	4	0.0029	4a	9	-0.153	33.30%
*S. fendleri*	*wild*	AABB	1	3	0.0048	3	12	ND	58.30%
*S. braschistotrichum*	*wild*	BB	2	4	0.0052	4	16	-0.07	68.80%
*S. sparsipilum*	*wild*	AA	2	4	0.0068	4	21	-0.108	57.10%
*S. spegazzinii*	*wild*	AA	7	13	0.0072	13b	55	-1.4	47.30%
*S. stoloniferum*	*wild*	AABB	2	7	0.0093	7	36	0.286	69.40%
*S. cardiophyllum*	*wild*	BB	2	4	0.0099	4	29	0.445	41.40%
*S. gourlayi*	*wild*	AAAA	2	8	0.0109	8	55	-0.789	50.90%
*S. vernei*	*wild*	AA/AAAA	6	16	0.0110	15bc	61	-0.148	42.60%
*S. tuberosum* ssp *andigena*	*cultivated*	AAAA	12	46	0.0121	39a	120	-1.177	56.70%
*S. tuberosum* ssp *tuberosum*	*cultivated*	AAAA	4	16	0.0124	14c	74	-0.112	45.90%
*S. polytrichon*	*wild*	AABB	1	3	0.0124	3	31	ND	64.50%
*S. stenotomum*	*cultivated*	AA	2	4	0.0137	4	43	-0.275	46.50%
*S. bulbocastanum*	*wild*	BB	1	1	-	1	ND	ND	
**Total**				**147**	**0.0134**	**133**		**all NS > 10%**	**60.50%**

Acc: accessions, seq: sequence, NS:non-synonymous, ND : not determined.

Three pairs of species (a: *S. tuberosum* ssp *andigena*/*S. phureja*, b: *S. vernei*/*S. spegazzinii and c: S. tuberosum* ssp *tuberosum*/*S. vernei*) had one common haplotype.

* Genome formulae proposed by Matsubayashi [25].

### Phylogenetic analyses

**Figure 1:**
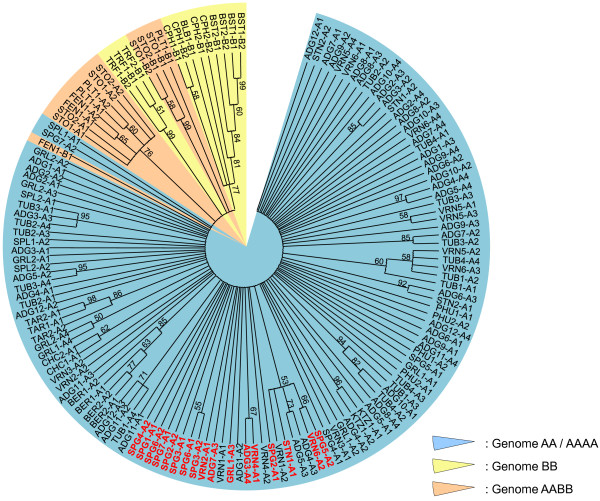
**Phylogenetic analysis of *Solanum *section Petota *RanGAP2 *sequences using maximum likelihood methods.** Phylogenetic analyses were conducted on the entire *RanGAP2* sequence dataset with 1000 bootstraps. All branches with bootstraps lower than 50% were collapsed. Genome formulae AA or AAAA, BB and AABB of the species studied are reported according to Matsubayashi [25] and highlighted by colored areas. The PF variants which present a proline residue at position 106 and phenylalanine residue at position 237 are represented in red.

Whatever the method used (maximum likelihood, maximum parsimony or minimum evolution), no clear evidence of phylogenetic structure was observed, as illustrated with the maximum likelihood analysis (Figure 1). Thus sequences from species with the genome formulae AA (diploid) or AAAA (tetraploid) were not structured according to the species origin. Nonetheless sequences derived from the diploid species with the genome formula BB [25] and from the di- and tetraploid species from Mexico and the USA tend to group together.

**Figure 2:**
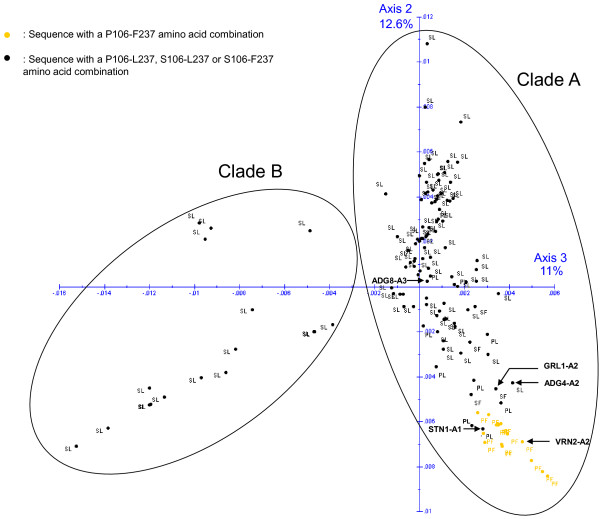
**Factorial analysis based on the *RanGap2 *dissimilarity matrix.** Results of the factorial analysis are shown in a two axis system representing 23.6% of the variability (11% for the horizontal axis and 12.6% for the vertical axis). Each point corresponds to one *RanGap2* sequence: the nature of amino acids 106 and 237 is indicated for each sequence. The distance between two points represents the dissimilarity value between these two sequences in the two axis representation. The two circles cluster all sequences of “clade A” and “clade B”. An arrow indicates the position of each *RanGAP2* variants used for transient expression study.

Findings from the phylogenetic tree were then confirmed with factorial analysis. As can be seen in Figure 2, axis 2 and axis 3 explained 12.6% and 11% of the total observed variability, respectively, based on the *RanGAP2* dissimilarity matrix. The dataset was divided into the two clades, A and B according to the genome formula, the clade A correspond to the species with the genome AA or AAAA. The lack of structure in “clade A” may result partially from the high variability observed within accessions. Analysis of molecular variance (AMOVA) showed that the intra-accession variability explained more than 67% of the total variability; variability observed among species and among geographical origin groups (Peru, Argentina, Bolivia, Colombia, Mexico, USA and Europe) explained 26.62% and 12.82% of the total variability, respectively (Table 2).

**Table 2:** AMOVA of the RanGAP2 dataset using two different partitioning criteria: species or geographic origin

**(A)**						
**Source of variation**			**Degree of freedom**	**Sum of squares**	**Variance components**	**Percentage of variation**
**Among groups**			17	530.2	3.17	26.62
**Among genotypes within groups**			37	372.9	0.72	6.02
**Within genotypes**			93	745.2	8.01	67.36
**Total**			147	1648.3	11.9	
**(B)**						
**Source of variation**			**Degree of freedom**	**Sum of squares**	**Variance components**	**Percentage of variation**
**Among groups**			6	232.607	1.49236	12.82
**Among genotypes within groups**			41	554.571	2.09563	18
**Within genotypes**			79	636.483	8.05675	69.18
**Total**			126	1423.661	11.64474	

The Analysis of MOlecular Variance (AMOVA) was carried out considering (**A**) 18 *Solanum* species or (**B**) seven geographical origins (Peru, Argentina, Bolivia, Colombia, Mexico, USA, Europe). In each case, the variance was calculated among the different groups, among the genotypes within each group and within all genotypes. In the AMOVA considering geographical origin, 21 sequences with an unknown geographical origin were removed from the dataset.

### Identification of positions of functional relevance in *RanGAP2*

Despite the low nucleotide diversity (1.3%) observed along the *Solanum RanGAP2* sequence dataset, we previously noted that nearly 50% of the mutations observed correspond to non synonymous mutations. Overall, 178 positions distributed along the entire protein sequence appear to be affected by non synonymous mutations, however only 28 of them appear to affect more than two *Solanum* species and 19 out of these 28 (*ie* nt positions 98, 175, 191, 309, 316, 709, 712, 787, 965, 1056, 1112, 1122, 1129, 1198, 1201, 1316, 1340, 1401, 1427) are located in either the WPP or LRR domains.

To identify functionally relevant variants among these 19 positions, we investigated whether evidence for positive selection pressure could be detected in our data, and carried out evolutionary analyses using either neutrality or dN/dS tests. Neutrality tests (Tajima’s D) were conducted at the intra-specific level in each of the investigated species, while dN/dS tests were conducted at the inter-specific level using SLAC, FEL and PAML (M1, M2, M7 and M8 models) methods. Values showing an excess of rare variants were obtained for the Tajima’D statistic (Table 1), but none of these neutrality tests provided significant results that allow a clear distinction from neutrality. In dN/dS methods, first using a reduced sequence dataset consisting of only one sequence per *Solanum* species (ie 18 *RanGAP2* aligned sequences), we compared the four evolutionary models implemented in the CODEML program: M1 vs M2 and M7 vs M8. None of the positive selection models appeared to be better adapted than the null model (2Δl = 9.84; NS p = 0,001). However, using the total sequence dataset (the 147 *RanGAP2* aligned sequences), the positive selection models appear better adapted (2Δl > 69.28; p < 0.001) and both the M2 and M8 models founded eight sites with dN/dS values significantly > 1 (Table 3). The polymorphisms at amino acid positions 106 and 237 were also identified by the FEL and/or SLAC methods (Table 3). However, SLAC and FEL also revealed, respectively, 38 and 52 sites significantly (posterior probability > 95%) under negative (purifying) selection suggesting that *RanGAP2* is not a gene evolving under positive selection.

**Table 3:** **Analysis of *RanGAP2 *for positively selected sites**

		**Used methods for detecting sites of interest**			
**Animo acid position**	**Domain in the RanGAP2 gene**	**M2**	**M8**	**FEL**	**SLAC**
33	WPP	0.999**	1.000**	Not identified	Not identified
106	WPP	0.999**	1.000**	0.992*	0.972*
165	LRR	0.957*	0.991**	0.937	0.938
237	LRR	0.999**	1.000**	0.991*	0.901
238	LRR	0.961*	0.988*	Not identified	Not identified
377	LRR	0.976*	0.995**	Not identified	Not identified
447	LRR	0.985*	0.995**	0.927	
519		0.999**	1.000**	Not identified	Not identified

Using a dataset comprising both intra- and inter-specific sequences, probabilities of eight sites of *RanGAP2* being under positive selection according to the M2 and M8 models of the CODEML program of PAML (Phylogenetic Analyses by Maximum Likelihood) and the FEL (Fixed Effects Likelihood) and SLAC methods (Single Likelihood Ancestor Counting), both available through the DataMonkey web interface.

It appears from these analyses that only two sites of interest (P/S 106 located in the WPP domain and F/L 237 located in the LRR domain) can be highlighted from the 19 sites previously identified. Furthermore, positions 106 and 237 were in linkage disequilibrium (D’ value of 0.766) [26,27]). A proline residue at position 106 was found to be significantly more frequently associated with a phenylalanine residue at position 237 than with a leucine residue (exact Fisher test and chi-square test) (Table 4). The amino acid combination P106-F237 was identified in 77.9% of the *S. spegazzinii* sequences (10/13), 25% of the *S. stenotomum* sequences (1/4), 18.7% of the *S. vernei* species (3/16), 12.5% of the *S. gourlayi* sequences (1/8) and in 4.3% of the *S. tuberosum* spp *andigena* sequences (2/46). These five species belong to “clade-A”. Despite the lack of structure within “clade-A”, these 17 sequences all clustered close to each other (Figure 2) and correspond to 14 *Solanum* accessions that all belong to the Hawkes series of wild Tuberosa or cultivated Tuberosa. This probably explains the phylogenetic proximity of these 17 sequences with the amino acid combination P106-F237.

**Table 4:** **Linkage disequilibrium between residues at positions 106 and 237 in *RanGAP2***

**(A)**		
**haplotype 106-237**	**Observed**	**Expected**
**P-F**	17	4
**P-L**	10	23
**S-F**	4	17
**S-L**	116	103
**(B)**		
**D’**	**Fisher test**	**Chi-square test**
0.766	p<0.001	p<0.001

Theoretical combinations of proline (P) and serine (S) at position 106 with phenylalaline (F) and leucine (L) were calculated using the frequency of each amino acid in the total dataset and assuming that the nature of an amino acid at position 106 is not linked to the nature of an amino acid at position 237. These theoretical combinations were compared to the observed combinations in the dataset (A). Linkage disequilibrium between residues at positions 106 and 237 was evaluated using the D’ value, the significance of which was tested with Fisher and Chi-square tests (B).

**Figure 3:**
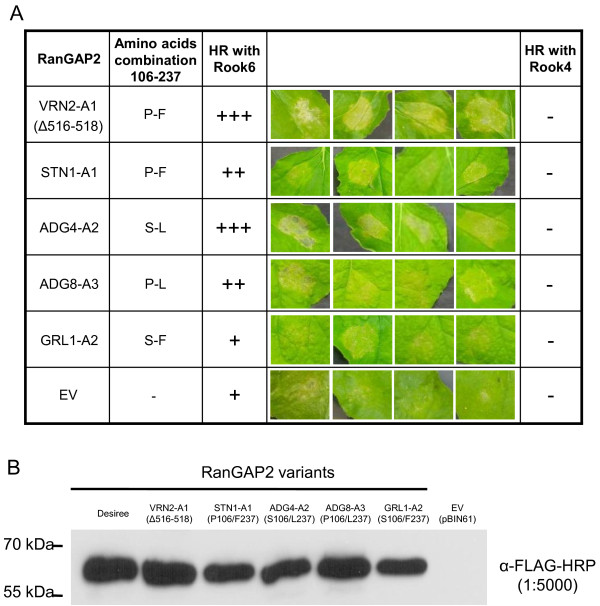
***RanGAP2 *variability affects the strength of the *Gpa2*-mediated HR in *Nicotiana benthamiana *leaves.** (**A**) The five *RanGAP2* variants and an empty vector (EV) were transiently expressed in *N. benthamiana* leaves with *Gpa2* and either the *Gp-Rbp-1 Rook-6* and *Rook-4* variants*.* The strength of the hypersensitive response obtained for each *RanGAP2* variant is indicated as follows: (+++) complete collapse and rapid desiccation of the infiltration patch within two days, (++) complete collapse of the infiltration patch by three days post-infiltration, (+) slow and incomplete collapse with residual live cells. (**B**) Immunoblot with horse radish peroxidase-conjugated anti-FLAG antibody demonstrating relative protein levels of the five transiently expressed RanGAP2 proteins. These five *RanGAP2* variants include VRN2-A1 which has a three amino acid deletion (Δ516-518), STN1-A1 (P106 and F237), ADG4-A2 (S106 and L237), ADG8-A3 (P106 and L237) and GRL1-A2 (S106 and F237) which represent the four haplotype combinations at the 106 and 237 positions. An empty vector (EV) was used as a negative control.

In order to test whether the P/S 106 and/or F/L 237 polymorphisms affect the function of GPA2, we tested whether RanGAP2 variants harboring different combinations at these two positions might allow GPA2 to gain recognition of previously unrecognized versions of GP-RBP-1 and/or allow it to work more efficiently. Five variants of *RanGAP2* were used in our experiments: VRN2-A1 (Δ516-518), which has a three amino acid deletion; STN1-A1 (P106 and F237), ADG4-A2 (S106 and L237), ADG8-A3 (P106 and L237) and GRL1-A2 (S106 and F237). Each *RanGAP2* variant was transiently expressed in *N. benthamiana* leaves with *Gpa2* and the Rook-4 (unrecognized) or Rook-6 (recognized) *Gp-Rbp-1* variants. In all cases, a HR was obtained when Rook-6 was expressed with *Gpa2* together with one of the five *RanGAP2* variants (Figure 3A). A weak HR was observed when the empty vector (EV) is used most probably due to the endogenous tobacco RanGAP2 that remains. No HR was observed when Rook-4 was co-expressed with *Gpa2* alone or with *Gpa2* in combination with any of the tested *RanGAP2* variants (Figure 3A). Thus no gain of recognition of Rook-4 by *Gpa2* was conferred by polymorphisms observed at positions 106 and 237 or by the three amino-acid deletion in RanGAP2. When using *CP* variants of PVX and *Rx* (instead of *Gp-Rbp-1* variants and *Gpa2*) with the same five *RanGAP2* variants similar results were obtained (ie an HR was observed with the recognized “TK” CP variant expressed with *Rx* but no HR was obtained for the “KR” CP variant, regardless of the *RanGAP2* variants used) (data not shown). Thus, natural variation at positions 106 and 237 also has no effect on *Rx* recognition of the “KR” variant of the PVX CP*.*

Despite the detection by immunoblotting of similar RanGAP2 levels of all variants (Figure 3B) it appeared that the intensity of the HR observed with the positive control Rook-6 varied depending on the *RanGAP2* variant expressed. Indeed, the HR obtained in transient *Agrobacterium* transformations with variant ADG4-A2 (S106 - L237) and VRN2-A1 (Δ516-518) was consistently stronger than that obtained with the three other *RanGAP2* variants (Figure 3A). It thus appeared that the most common amino acid combination S106-L237 seemed to be the most effective at triggering the HR, which ensued from the recognition of avirulent *Gp-Rbp-1* Rook-6.

## Discussion

### *RanGAP2*: a conserved gene involved in plant-pathogen interactions

*Solanum RanGAP2* variants appear to show a high degree of conservation. With the exception of only one of the 147 *RanGAP2* sequences obtained in this study (which showed a three amino acid in-frame deletion in the N terminus), all others have the same length (1662 bp ORF) and are characterized by a WPP domain at the 5’end (corresponding to amino acids 14 to 109) and ten LRRs of 28 amino acids each. Their overall structure is also well conserved among the 55 accessions of the 18 *Solanum* species studied; the two most divergent alleles (BST1-B2 and TUB2-A3) are still 97% identical at the nucleotide level.

*RanGAP2* shows less sequence size variations and a lower polymorphism rate (31.4% vs 19%) than *Gp-Rbp-1*[20], the gene encoding for the effector recognized by GPA2. Furthermore *RanGAP2* also appears to be less variable than the housekeeping gene nitrate reductase, whose variability was evaluated in various *Solanum* species [28]. The estimated pi value for two *S. stoloniferum* accessions (eight sequences) and one *S. sparsipilum* accession (two sequences) is at least twice as high for the nitrate reductase gene [28] than that estimated in these two species for the *RanGAP2* gene (two *S. stoloniferum* and one *S. spegazzinii* accession were also represented in our dataset).

The phylogenetic tree generated with the 147 *RanGAP2* sequences correlates well with several phylogenies obtained with neutral markers or using DNA sequences of single copy housekeeping and chloroplast genes [28-32]. We can also note that *RanGAP2* sequences of the three allo-tetraploid species *S. stoloniferum*, *S. fendleri* and *S. polytrichon* are phylogenetically related. This is consistent with the study of Jacobs *et al*., [31] which considered these three taxa as a single species. Our *RanGAP2* dataset also confirms a lack of phylogenetic structure in *Solanum* section Petota partly attributed to numerous interspecific hybridizations at both the diploid and polyploid levels [28]. This can explain how three pairs of species (*S. tuberosum* ssp *andigena*/*S. phureja*, *S. tuberosum* ssp *tuberosum*/*S. vernei* and *S. vernei*/*S. spegazzinii*) have one *RanGAP2* haplotype in common.

Positive selection hallmarks in *RanGAP2* were searched using the Tajima’s D statistic. Though negative values were mostly obtained for the species investigated, none appear significantly different from neutral expectations. Positive values of the Tajima’s D statistic were observed in *S. cardiophyllum* (genome BB) and its sister derived species *S. stoloniferum* (genome AABB). It is tempting to speculate that the traces of balancing selection can be linked to the BB genome. However calculation of the values of the Tajima’s D statistic was hampered in most of the other species corresponding to the BB genome by the lack of at least four different alleles. We also took advantage of the sequencing of RanGAP2 in multiple species to investigate the evolutionary constraints acting on this gene based on dN/dS ratios. Using a reduced dataset made of one consensus sequence per species in order to consider only inter-specific sequences as is recommended [33-35], *RanGAP2* does not appear to be under positive selection and appears rather to be under strong evolutionary constraints as previously suggested by its low diversity and as also confirmed by the numerous (at least 38) sites under purifying selection identified by both SLAC and FEL analysis. However, using the complete sequence dataset we were able to identify two key positions (aa position 106 and 237) that are predicted by at least two different methods to be under positive selection. One possible explanation of this pattern is that alleles displaying these two amino acid changes, also found in linkage disequilibrium, have passed through the different *Solanum* species as an advantageous combination. In a similar way, key residues under positive selection were detected in the translation factor eIF4E, which is also involved in resistance to potyviruses in pepper, using a data set comprising both intra- and inter-specific sequences [36,37]. Similarly to eIF4E, the two key positions identified in RanGAP2 using the whole RanGAP2 dataset could be linked to its role in the incompatible resistance interaction between CP and Rx or Gp-RBP-1 and GPA2.

### Impact of *RanGAP2* polymorphism on plant-parasite interactions

Although GP-RBP-1 has not been definitively shown to physically interact with RanGAP2, this protein is thought to play a role in mediating recognition of both PVX CP and GP-RBP-1 via the LRRs of Rx and GPA2, respectively, as proposed by the bait and switch model and by artificial tethering experiments [5,19,38]. Potential for variation in effector recognition due to variation in bait proteins is suggested from studies of the tomato NB-LRR Prf, which recognizes Avr proteins from *Pseudomonas syringae* through its cofactor, the Pto kinase [21]. Multiple Pto-like proteins are encoded in the tomato genome, and it has been shown that Prf recognition specificity is altered depending on which homologue it interacts with [39,40]. As such, variability in RanGAP2 could affect GPA2 – RBP1 interactions. Sites under positive selection are often key positions in resistance interactions and, more specifically, in the recognition of Avr proteins [19,36,41]. We tested if the nine nucleotide deletion in the N-terminus of *RanGAP2* and/or the variability observed at sites 106 and 237 could alter or enhance GPA2-mediated recognition of GP-RBP-1 by transient expression in tobacco leaves.

The GP-RBP-1 “Rook-4” was never recognized by GPA2 regardless of which *RanGAP2* variants were co-expressed. This is consistent with previous results showing that although different GP-RBP-1 variants elicited GPA2-dependent HRs to varying degrees, ultimately, polymorphism at position 187 of GP-RBP-1 [19] was the only variation which could explain GPA2-mediated recognition of GP-RBP-1. Similar results were obtained on the recognition of the CP variant “KR”, which is recognized only very poorly by Rx [42,43]. As a weak HR is obtained with the empty vector, these results should be interpreted carefully because in addition to the *RanGAP2* variants tested endogenous *N. benthamiana RanGAP2* are expressed in the agro-infiltrated leaves and could affect the interaction.

Sites under positive selection in the LRR domains of R genes do not always localize to the R/Avr interface [41,44]. In addition some amino acids in the LRR domain modulate the resistance by interacting with host factors [45]. Thus, diversity observed at RanGAP2 sites 106 and 237 (sites detected with software usually used to find positive selection) and the three amino acid deletion (Δ516-518) may affect the resistance interaction through a mechanism other than recognition. Position 106 localizes to the WPP domain, which physically interacts with the CC domain of both Rx and GPA*2*[16,19,46] which has recently been shown to be sufficient to activate Rx fragments upon co-expression [22]*.* Diversity at position 106 could thus affect the three-dimensional conformation of the entire GPA2/RanGAP2/GP-RBP-1 complex and thus contribute to the initiation of defense mechanisms by facilitating transition from an auto-inhibited to an active form of the NB-LRR protein [5].

Our results also resemble those recently obtained in tomato on the decoy RCR3 for which the functional assay of several alleles shows that the polymorphisms observed do not play a role in pathogen (*C. fulvum*) recognition but are responsible of the modulation of defence response upon effector recognition [13]. However, while *RCR3* was characterized by balancing selection, *RanGAP2* experienced rather purifying selection. This difference in rate of evolution can be related to the difference of either the variation in time and space of the selection pressure exerted by nematodes vs fungus or the ability of these two co-factors to interact physically with pathogen effectors. Indeed, RCR3 is able to bind to AVR2 proteins of the fungus [47] and was also showed recently to be able to bind to a nematode effector [48] while it is still unclear if RanGAP2 is able to bind GP-RBP-1. Artificial tethering of RanGAP2 and GP-RBP-1 through YFP complementation allowed these two proteins to interact physically but did not lead to the recognition of “Rook-4” by *Gpa2*[19]. On the other hand, tethering of RanGAP2 to a GP-RBP-1 with a proline residue at position 187 (recognized by GPA2) enhances *Gpa2*-mediated HR [19]. Consequently, it appears that the P/S-187 position of GP-RBP-1 qualitatively determines whether GPA2 will recognize RBP-1, whereas the RanGAP2 positions 106 and/or 237 and the three amino acid deletion (Δ516-518) may enhance the efficiency of this recognition, resulting in a faster and more intense HR.

Whether the strength of this recognition is a consequence of only the GPA2 + RanGAP2 physical interaction or also that of a RanGAP2 + GP-RBP-1 physical interaction remains to be elucidated. However, the observed rate of evolution in RanGAP2 appears to be not in support of a potential physical interaction with so divergent and polymorphic nematode and virus effectors. It seems rather that the different variants of RanGAP2 allow *Rx* or *Gpa2* to function more efficiently independent of the recognition of the pathogen effector. This would predict that the diversity of recognition cofactors represents an additional level of complexity in plant-pathogen interactions that merits further study.

## Conclusions

In this study sequence variability of the ETI cofactor RanGAP2 was investigated for the first time and the potential of variants of this gene to enhance resistance to nematodes or viruses was examined. We identified a three amino-acids deletion and two sites (one in the WPP domain and one in the LRR domain) that should help to define variants of interest. Variability observed in theses sites/region of RanGAP2 does not seem to enable recognition of virulent variants of GP-RBP-1 by GPA2 but appears to enhance the recognition of avirulent variants of GP-RBP-1.

## Methods

### Plant materials, DNA isolation, amplification and cloning

*Nicotiana benthamiana* seeds were germinated and plants were grown for 5 weeks in a Conviron growth cabinet (Conviron, http://www.conviron.com), where conditions were as follow: 21°C/21°C day/night, 16-h day, 60% relative humidity, and light intensity of 100 μmol m^-2^ s^-1^.

Fifty five diploid and tetraploid accessions from 18 *Solanum* species belonging to the plastid DNA clade 2 and 4 of section Petota [29] were examined (Table 5). Total DNA from each accession was isolated using the protocol described by Fulton et al. [49]. A Genbank *RanGAP2 S. tuberosum* sequence (http://www.ncbi.nlm.nih.gov/nuccore/AM411448) was used to design primers in the 5’ and 3’ ends of the 1662pb intronless gene. PCR reactions were carried out in a final volume of 25 μL using 30 ng of template genomic DNA, 0.5 μM of the RanGap2JC1-F forward primer (5’-ATGGATGCCACAACAGCTAA-3’), 0.5 μM of the RanGap2stop reverse primer (5’-CTAATTGCTATCTGGTGTGTCAAG-3’), 0.2 mM of each dNTPs, 0.6 unit of Taq polymerase (Takara Ex Taq™), and 2.5 μL of the provided 10× *Ex Taq* Buffer. The PCR protocol was 5 min at 98°C, followed by 35 cycles of: 10 sec at 98°C, 30 sec at 51°C and 1 min 45 sec at 72°C and a final step of 10 min at 72°C.

**Table 5:** **Detailed informations on the *Solanum *species analyzed in this study**

							***RanGap2 *106–237 haplotype**			
**Species (classification reviewed by Jacobs *et al.*, 2008)**	**Genotype code**	**Accession**	**Ploidy level**	**Country of origin**	***G. pallida Rce***	**PVX Rce**	**PF**	**PL**	**SF**	**SL**
*S. tuberosum spp. andigena*	ADG1	88S.233. 8	4×	Peru	---	+++		x		x
	ADG2	88S.249. 1	4×	Bolivia	---	+++				x
	ADG3	88S.250. 1	4×	Argentina	---	+++	x	x		x
	ADG4	88S.262. 7	4×	Peru	---	---		x		x
	ADG5	88S.408. 14	4×	-	--- (**)	+++		x		x
	ADG6	88S.257. 4	4×	Peru	---	---				x
	ADG7	88S.259. 9	4×	Peru	---	---	x			x
	ADG8	88S.260. 11	4×	Peru	---	---		x		x
	ADG9	88S.261. 3	4×	Peru	---	+++				x
	ADG10	88S.263. 4	4×	Peru	---	+++				x
	ADG11	88S.264. 7	4×	Peru	---	---				x
	ADG12	88S.255. 2	4×	Mexico	---	---				x
*S.berthautii*	BER1	88S.282.8	2×	Bolivia	---	---				x
	BER2	88S.452.8	2×	-	---	+++				x
*S. bulbocastanum*	BLB1	00S. 32. 11	2×	Mexico	---	---				x
*S. brachistotrichum (S. stenophyllidium)*	BST1	00S. 17. 5	2×	Mexico	---	---				x
	BST2	00S. 19. 1	2×	Mexico	---	---				x
*S. chacoense*	CHC1	88S.456.8	2×	Argentina	--- (*)	---				x
	CHC2	74S. 33. 3	2×	?	?	?				x
*S. cardioophyllum*	CPH1	00S. 42. 3	2×	Mexico	---	---				x
	CPH2	00S. 43. 21	2×	Mexico	---	---				x
*S. fendleri (S. stoloniferum)*	FEN1	00S. 56. 1	4×	USA	---	---				x
*S. gourlayi*	GRL1	88S.315.18	4×	Argentina	---	+++	x		x	x
	GRL2	88S.495.5	4×	Bolivia	+++	+++				x
*S. kurtzianum*	KTZ1	88S.499. 10	2×	Argentina	+++ (*)	---				x
*S. phureja*	PHU1	88S.214.14	2×	Colombia	---	---				x
	PHU2	78S.222. 8	2×	Peru	---	+++				x
*S. polytrichon (S. stoloniferum)*	PLT1	00S. 69. 8	4×	MEXICO	---	---				x
*S. spegazzinii*	SPG1	78S.236. 2	2×	Argentina	+++	---	x			
	SPG2	88S.332.2	2×	Argentina	+++	---	x			
	SPG3	88S.334.19	2×	Argentina	+++	---	x			
	SPG4	88S.510.9	2×	Argentina	+++	---	x		x	
	SPG5	88S.511.7	2×	Argentina	+++	---	x		x	
	SPG6	88S.514.3	2×	Argentina	+++	---	x			
	SPG7	88S.524.24	2×	Argentina	+++	---	x			x
*S. sparsipilum*	SPL1	88S.329.18	2×	Bolivia	+++	---				x
	SPL2	99S. 74. 9	2×	Peru	---	---				x
*S. stenotomum*	STN1	74S. 14. 1	2×	Peru	---	---	x			x
	STN2	74S. 16. 3	2×	Bolivia	---	---				x
*S. stoloniferum*	STO1	69S.107. 15	4×	?	?	?				x
	STO2	00S. 83. 13	4×	Mexico	---	---				x
*S. tarijense*	TAR1	90S. 6. 4	2×	Argentina	---	---				x
	TAR2	90S. 14. 31	2×	Argentina	---	---				x
*S. trifidum*	TRF1	00S. 99. 3	2×	Mexico	---	---				x
	TRF2	00S.100. 20	2×	Mexico	---	---				x
*S. tuberosum ssp. tuberosum*	TUB1	Desiree	4×	Europe	---	---				x
	TUB2	Darwina	4×	Europe	+++ (**)	+++				x
	TUB3	Multa	4×	Europe	?	++				x
	TUB4	Glenna	4×	Europe	++ (**)	+++				x
*S. vernei*	VRN1	74S. 32. 1	2×	?	+++	?		x	x	
	VRN2	78S.248. 1	2×	Argentina	+++	---	x			x
	VRN3	88S.342.5	2×	Argentina	+++	---				x
	VRN4	88S.530.14	2×	Argentina	+++	---	x	x		
	VRN5	AM 78 3778	4×	?	+++	?				x
	VRN6	SCRI 12380	4×	?	+++	?	x			x

The species and country of origin of the 55 accessions used in this study are detailed in the table. The genotype code used in this study is indicated with the corresponding accession number of the considered genotype. *G. pallida* and PVX resistance (Rce) information is also included for each genotype. Resistance to *G. pallida* may have been evaluated only for pathotype 3 (*) or pathotype 2 (**). These informations were obtained from either the Sturgeon Bay collection, Braunschweig Institute or INRA. The amino acid at position 106 and 237 for each *RanGAP2* sequences obtained are also indicated in the table.

PCR products were cloned using the Strataclone™ PCR cloning Kit (Stratagene) according to the manufacturer’s instructions. Ten transformed colonies per tetraploid accession and five per diploid accession were selected and used to amplify the insert. PCR reactions were performed in a final volume of 60 μL, containing 0.5 μM of M13 forward and reverse primers, 0.2 mM of each dNTPs, 1.5 unit of Taq polymerase (Takara Ex Taq™) and 6μL of the 10× *Ex Taq* Buffer. The PCR protocol was 5 min at 98°C, followed by 35 cycles of 10 sec at 98°C, 30 sec at 55°C, 2 min 30s at 72°C and a final step of 10 min at 72°C. PCR products were sequenced by Genoscreen using the following three primers: RanGap2JC695-R reverse primer (5’ CTAACTCCCTTCTCACCCAGA 3’) for the 3’end sequence, RanGap2JC435-F forward primer (5’ GCCATTAAAAGAGCCTGGAA 3’) for the 5’ end sequence and RanGap2JC1015-F forward primer (5’ GGTCCAGAAGTTGGTCTTGTGT 3’) as an internal sequencing primer. All the sequences have been deposited to GenBank/EMBL databases under the following accession numbers HE681572 to HE681718. For each RanGAP2 allele, the EMBL number accession is reported in Additional file 1: Table S1.

### Sequence alignments and evolutionary analyses

Previously obtained sequences were assembled using the cap contig assembly program of Bioedit [50]. This same module was used to construct the consensus sequence of *RanGAP2* for each species. When possible, Mega5 software was used to manually correct ambiguous nucleotides based on the chromatograms [51]. *RanGAP2* sequences were aligned using Mega5 [51] and the ClustalW (1.6) DNA weight matrix. Mega5 [51] was also used to find the best DNA substitution model fitting our dataset. Phylogenetic analyses on *RanGAP2* sequences were carried out using, maximum likelihood and maximum parsimony methods and minimum evolution algorithms with the Tamura 3-parameter model [52] and a Gamma parameter of 0.163. This model was the best model fitting our dataset and available in Mega5 software [51]. The robustness of the minimum evolution trees was evaluated by bootstrapping with 1000 repetitions. Motifs were detected in translated DNA sequences using SMART (Simple Modular Architecture Research Tool) [53]. The pi indicator (average number of nucleotide differences per site between two sequences), haplotype diversity was computed using DnaSP v5 software [54].

To examine whether *RanGAP2* has evolved under positive selection we used the ratio of non-synonymous to synonymous substitution rates per site (ω=dN/dS) estimated by site specific models implemented in the PAML package v 3.14 [55]. The tree used in PAML analysis was first obtained after running M0 in PAML (model = 0 and NS sites = 0. The CODEML program of PAML assigns a likelihood score to models for selection. A likelihood score for a model incorporating positive selection (M2 or M8) that is higher than that for a null model without positive selection (M1 or M7) is evidence for positive selection. To identify the sites under positive selection we used several different methods including the Bayes Empirical Bayes implemented in CODEML, which calculates the posterior probabilities that each site fell into a different ω class, the Single-Likelihood Ancestor Counting (SLAC) [56] and the Fixed-Effects Likelihood (FEL) [56], both available through the DataMonkey web interface [57]. Linkage disequilibrium values between sites of interest were evaluated by calculating the “ D’ ” value associated with its Fisher and Chi-square statistical tests, using DnaSP v5 software [54]. The intra- and inter-species/genotype variabilities were evaluated by AMOVA using Arlequin 3.1 software [58].

### Agrobacterium transient transformation assays

The five *RanGAP2* sequences STN1-A1 (P-106, F-237), ADG4-A2 (S-106, L-237), ADG8-A3 (P-106, L-237), GRL1-A2 (S-106, F-237) and VRN2-A1 (Δ516-518) were chosen to represent all the variability observed at the two sites 106 and 237. To generate *RanGAP2* expression clones, the different inserts were ligated into the 5’ *Xba*I and 3’ *Bam*HI sites of the pBIN61 binary vector series. For this, the five *RanGAP2* sequences were amplified with forward primer RG2×ba (5’-AGTCTAGAACCACCATGGATGCCACAACAGCTAA-3’) and reverse primer RG2Bam (5’ATGGATCCATTGCTATCTGGTGTGTCAAGA-3’) to add *Xba*I and *Bam*HI restriction sites at 5’ and the 3’ ends of the PCR products. After purification on agarose gel, amplicons were first ligated into pGEM®-T (Promega Easy Vector Systems), then digested with *Xba*I and *Bam*HI and ligated into pBIN61 vector. An empty vector was used as a negative control. The “Rook-4”, “Rook-6”, “KR” and “TK” expression clones were obtained as previously described [19,59]. For transient protein expression, *N. benthamiana* plants were infiltrated by syringe with *Agrobacterium tumefaciens* strain C58C1 carrying the virulence plasmid pCH32 and the appropriate pBIN61 binary expression vector. *Agrobacterium* cultures were diluted to OD600 = 1 and co-infiltrated at a final OD600 = 0.33 (OD600=0.2 for western blotting). Plants were transferred to a growth chamber maintained with 16-h light and 8-h darkness at 20°C for three to five days. All experiments were repeated on at least three leaves of three different plants.

### Protein extraction and western blotting

For protein extractions, leaf discs were grounded in liquid nitrogen and resuspended in 70 μl of 1× sodium dodecy sulphate (SDS)–PAGE loading dye. Samples were heated at 95°C for three minutes and centrifuged at 18 000 × g to get rid of cell debris. 35 μl of cleared protein lysate was loaded on a 10.5% acrylamide gel and separated by SDS–PAGE electrophoresis. Proteins were then transferred to polyvinylidene difluoride (PVDF) membranes (Bio-Rad, http://www.biorad.com) and blots were blocked for 90 min with Tris-buffered saline (TBS) containing 5% (w/v) powdered skimmed milk and 0.1% (v/v) Tween 20. For detection of RanGAP2 variants, blots were probed for 1 hour with horseradish peroxidase-conjugated anti-FLAG (M2; Sigma, http://www.sigmaaldrich.com) antibodies diluted in TBS plus 1% (w/v) powdered skimmed milk and 0.1% (v/v) Tween 20. Antibodies dilution was 1:5000. Blots were washed 4 times with TBS plus 0.1% (v/v) Tween 20 and epitope-tagged proteins were visualized using the ECL chemiluminescent system (Pierce, http://www.piercenet.com).

## Competing interests

The authors declare that they have no competing interests.

## Authors’ contributions

The study was conceived by JC, MJMD, MCK, PM and EG. JC obtained and analyzed the sequence dataset. JC, ME, and LPH carried out the transient expression assays and immunoblotting analyses. JC wrote the manuscript and MJMD, EG, LPH, PM and MCK proofread the manuscript. All authors read and approved the final manuscript.

## Supplementary Material

Additional file 1: Table S1 Detailed informations on the different RanGAP2 alleles analysed in this study. For each RanGAP2 allele analysed, species and accession of origin, nature of the amino acids 106 and 237 and RanGAP2 allele EMBL number accession are indicated in the table. Click here for file
